# Supplementary material for: Adaptive and migration-enhanced tree seed algorithm for multi-threshold CT image segmentation and lung cancer recognition
Source: PLoS One. 2026 Jan 16;21(1):e0333304. doi: 10.1371/journal.pone.0333304 (PMC12810928; doi:10.1371/journal.pone.0333304)
Supplement: Attachment — Submitted filename: Response to Reviewers.pdf [file pone.0333304.s001.pdf]

Response to Reviewers

Chenxi Li
Center for Artificial Intelligence,
Jilin University of Finance and Economics,
Changchun 130117, China
Email: 0314042200902@jlufe.edu.cn
August 15, 2025
To: The Academic Editor and Reviewers, PLOS ONE
Manuscript ID: PONE-D-25-33941
Manuscript Title: Adaptive and Migration-enhanced Tree Seed Algorithm for Multi-Threshold CT Image Segmentation and Lung Cancer Recognition
Dear Dr. Omran and esteemed Reviewers,

Thank you for your thorough review of our manuscript and for providing insightful comments and constructive suggestions. We appreciate the time and effort you have dedicated to our work. We have carefully considered all the points raised and have revised the manuscript accordingly. We believe the manuscript has been significantly improved as a result of these revisions.

Below is our point-by-point response to the comments. All changes have been highlighted in the manuscript file labeled 'Revised Manuscript with Track Changes'.

Responses to the Academic Editor

Journal Requirement 1: *Please ensure that your manuscript meets PLOS ONE's style requirements, including those for file naming.*

Our Response: Thank you for this reminder. We have carefully reviewed the PLOS ONE style templates and have revised the manuscript to ensure full compliance with all formatting and file naming requirements.

Journal Requirement 2: *Please ensure that you refer to Figure 1, 2, 3 and 8 in your text...*

Our Response: Thank you for pointing this out. We have checked the manuscript and confirmed that all figures, including Figures 1, 2, 3, and 8, are now correctly referenced in the main text.

Journal Requirement 3: *Please ensure that you refer to Table 9 in your text...*

Our Response: Thank you. We have added a reference to Table 9 in the main text to ensure it is properly linked.

Journal Requirement 4: *If the reviewer comments include a recommendation to cite specific previously published works, please review and evaluate these publications...*

Our Response: We have carefully reviewed all publications recommended by the reviewers. We have cited those that are relevant to our work, including the seminal paper on the No Free Lunch theorem by Wolpert and Macready, and the critiques on metaheuristics by Sörensen and Camacho-Villalón et al., to enrich our discussion.

Responses to Reviewer #1

Our Response: We sincerely thank you for this comprehensive and insightful feedback, which has been invaluable in improving the quality of our manuscript. We agree that a rigorous evaluation requires fair comparisons, transparent parameterization, and a clear justification of novelty. In response to your comments, we have undertaken a major revision of the manuscript.

1. **Conciseness and Structure:** We have streamlined the paper. To improve clarity and focus, we have been more selective with our figures (e.g., removing unnecessary stability analysis).
2. **Parameter Sensitivity Analysis:** We agree that the use of hard-coded parameters requires justification. We have now added a new dedicated subsection, **Section 5.3, "Parameter Sensitivity Analysis"**, to investigate the impact of AMTSA's key parameters. This analysis provides an empirical basis for the parameter values used in our study **(see Page 32)**.
3. **Fairness of Comparisons:** This was a critical point, and we have completely overhauled our experimental protocol to ensure fairness.
   (1) The stopping criterion for all algorithms has been uniformly set to a maximum number of fitness evaluations (MaxFEs), as is now explicitly stated in **Section 4.1, "Experimental Fundamentals" (Page 14)**.
   (2) We have adopted algorithm-specific parameter settings, including population sizes, based on recommendations from the original literature. These are now transparently detailed in the revised **Table 1 (Page 16)**.
   (3) The comparative algorithm suite has been updated to include top-performing CEC competitors as you suggested.
4. **Novelty and Distinction:** We have revised our **Contribution section** to address your concerns about novelty **(Page 3)**, detailing the significant methodological differences and unique contributions of AMTSA compared to our prior works.
5. **Source Code Availability:** We sincerely apologize for the issue with the source code link. To ensure reproducibility, the complete source code will be provided as **supplementary material** with our submission. The public link will become active once the paper is successfully published.

We are confident that these substantial revisions have addressed your concerns. We will now respond to each of your specific points in detail below.

(Specific Comments)

Point 1: NFLT Citation Reviewer's Comment: *"line 20... It would better to cite the original paper Wolpert, David H., and William G. Macready. 1997..."*

Our Response: Thank you for this excellent suggestion. We agree that citing the original paper is more appropriate. We have now replaced the previous reference with the seminal paper by Wolpert and Macready (1997), as shown in the **revised manuscript on Page 1, Line 19**.

Point 2: Redundancy in Motivations/Contribution Reviewer's Comment: *"line 48... Motivations and Contribution are partly redundant. You should be more concise..."*

Our Response: Thank you for your comment. We have reviewed these sections and agree that there was overlap. We have now revised and condensed the "Motivations" and "Contribution" sections to eliminate redundancy and present our arguments more

concisely and forcefully, as can be seen in the **revised manuscript (Pages 2-3)**.

Point 3: Defining Exploration/Exploitation Reviewer's Comment: *"line 83... Please, rigorously define what exploration and exploitation are... And then prove, at least experimentally, that this two measures can indeed be balanced thanks to your approach."*

Our Response: Thank you for highlighting this crucial point. To address this, we have made two significant revisions. First, under Section 5, "Analysis and discussion," we have improved the subsection "Exploration and Exploitation Analysis" (Page 21), where we rigorously define how we quantify exploration and exploitation based on population diversity, a widely accepted method. Second, we have expanded our experimental analysis to include a diverse set of representative functions (F1, F8, F10, and F25). The results, presented in the new Figure 11 (Page 22), now clearly demonstrate how AMTSA's mechanisms achieve a more effective and adaptive balance.

Point 4: Hard-coded parameters and Sensitivity Analysis (formula 16 & 18, 19) Reviewer's Comment: *"line 260 (formula 16) Why 10? Sensitivity analysis? lambda: sensitivity analysis? ... line 299 (formulae 18,19) Several hardcoded parameters like 0.2 etc. Sensitivity analysis? And why (1.2-0.2) and not just 1?"*

Our Response: Thank you for pointing out the need for justification of these parameters. We have now added a new dedicated subsection, Section 5.3, "Parameter Sensitivity Analysis" (Page 32), which includes a thorough analysis of the key parameters, including the divisor in formula (16) and the range parameters in formula (18). The results of this analysis provide an empirical basis for the values chosen in our study. Regarding the notation (1.2-0.2), we have used this standard formulation to clearly indicate the defined range of the parameter beta, which varies from a maximum of 1.2 to a minimum of 0.2.

Point 5: Comparison with other TSA Variants Reviewer's Comment: *"line 533... Comparative Experiment 1: ... Not very useful. As TSA is anyway not a good algorithm challenging it is easy, and you could/should compare to just the best ones of these variants. And I wonder why you don't compare to ATSA [26] or KATSA [34] which have partly the same authors."*

Our Response: Thank you for this valuable feedback. To address your concern, we have revised our comparison strategy. In our first comparative experiment (Section 5.2.1), we have now included our prior works, ATSA and KATSA, for a direct and transparent comparison. This allows us to situate AMTSA within our research trajectory and clearly demonstrate its unique advancements. We have retained the comparison with other TSA variants to provide a comprehensive picture for readers familiar with the TSA literature, but the main focus is now on the comparison with state-of-the-art algorithms in the second experiment.

Point 6: Fairness of Comparison with State-of-the-Art Algorithms Reviewer's Comment: *"line 569... As you use the CEC 2014 benchmark you have to compare at least to the two or three first winners of this competition. Not only L-SHADE... but also GaAPPADE and maybe MVMO-SH. I also suggest CMA-ES. ... I am not sure you respect the conditions to correctly use the CEC 2014 benchmark, for fair comparison..."*

Our Response: We sincerely thank you for these critical comments, which have led to

a complete overhaul of our experimental protocol to ensure fairness and rigor.

1. We have updated our comparative algorithm suite in Section 5.2.2 to include the highly competitive algorithms you suggested: GaAPPADE, MVMO-SH, and CMA-ES.
2. We have strictly adhered to the CEC 2014 guidelines. The stopping criterion for all algorithms is now uniformly set to MaxFEs = D x 10,000, as stated in Section 4.1, "Experimental Fundamentals".
3. We have adopted algorithm-specific parameter settings, including dynamic population sizing for L-SHADE, based on recommendations from their original publications. All parameters are now transparently detailed in the revised Table 1 (Page 16).

Point 7: Critique of Metaheuristic Metaphors Reviewer's Comment: *"About GWO you should read... And more generally you should consider Sörensen, K. (2015)..."*
Our Response: Thank you for directing us to this important body of literature. We have read the recommended papers and agree with the call for more scientific rigor. We have added a new paragraph in the **Contribution section of the Introduction (Page 3)**, citing these works. In this new paragraph, we explicitly frame the contribution of AMTSA not on its natural metaphor, but on its concrete, quantifiable, and novel computational mechanisms.
Point 8: Tone of Discussion for Segmentation Results Reviewer's Comment: *"line 748... Table 13 "Although AMTSA does not always perform the best..." A bit optimistic."*
Our Response: Thank you for this observation. We have revisited our discussion of the CT image segmentation results and have revised the text to adopt a more objective and balanced tone. We now more clearly acknowledge the trade-offs between the PSNR and SSIM metrics and provide a more conservative interpretation of the results **(see revised manuscript, Page 52, Line 882)**.
Point 9: Fairness of SVM Comparison Reviewer's Comment: *"line 866... Table 14, Although the results seem interesting there is no way to evaluate the fairness of the comparisons. Please specify all parameters values..."*
Our Response: Thank you. To ensure the fairness and reproducibility of our SVM-based recognition experiments, we have now added a detailed description of the experimental setup, including that all comparative optimization algorithms operated within a unified, predefined search space. The search range for the SVM's penalty coefficient C was set to [0.1, 100], while the range for the RBF kernel function parameter gamma was set to [0.001, 1]. This information has now been added to the manuscript on Page 38.
Point 10: Typos Reviewer's Comment: *"Typos: weibull => Weibull (several times)"*
Our Response: Thank you for catching this. We have corrected "weibull" to "Weibull" throughout the manuscript and have performed another thorough proofreading to correct any other typographical errors.

Responses to Reviewer #2
Our Response: We sincerely thank you for your specific and constructive comments. Your suggestions, such as conducting an ablation study, a runtime comparison, and

providing visual examples, have been crucial in enhancing the rigor and completeness of our study. We have carefully revised the manuscript according to all your suggestions and believe these changes have significantly improved its quality. We will now respond to each of your points in detail.

(Specific Comments)

Point 1: Benchmark Sets Reviewer's Comment: *"The proposed algorithm should be evaluated using one of the recent CEC benchmark sets, such as CEC 2021 or CEC 2022."*

Our Response: Thank you for this valuable suggestion. We completely agree that evaluation on newer benchmarks is an important step in verifying an algorithm's generalization capabilities. For this revision, our primary goal was to conduct an extremely rigorous and fair performance comparison against the specific suite of top-tier algorithms suggested by the reviewers (such as L-SHADE, GaAPPADE, and CMA-ES). The most well-documented and validated results for these key competitors are on the IEEE CEC 2014 benchmark. To ensure a direct, apples-to-apples comparison with these established results and to avoid introducing new variables by changing the test suite, we decided to focus the scope of the current study on CEC 2014 to draw the most solid comparative conclusions. We have added your suggestion to test on newer platforms like CEC 2021/2022 as a very important direction in our **"Conclusions and Future Work" section (Page 46)**.

Point 2: Ablation Analysis Reviewer's Comment: *"- Conduct ablation analysis for your algorithm."*

Our Response: Thank you for this excellent recommendation. We have now conducted a comprehensive ablation study to validate the contribution of each of the three novel components in AMTSA. A new subsection, **Section 5.4, "Ablation Study of AMTSA Components,"** along with the **new Figure 19 and its analysis**, has been added to the manuscript **(Pages 34-35)**. This analysis strongly demonstrates that removing any single component leads to a significant degradation in performance.

Point 3: Empirical Runtime Comparisons Reviewer's Comment: *"- Include empirical runtime comparisons (e.g., AMTSA vs. DE or JADE) to assess practical scalability."*

Our Response: Thank you. To evaluate the practical computational efficiency and scalability of AMTSA, we have added a new subsection, **Section 5.5, "Computational Efficiency and Scalability Analysis."** In this section, we measure and compare the average CPU time required for AMTSA and other representative algorithms to complete the same number of function evaluations (MaxFEs). The results and analysis are presented in the **new Table 9 (Pages 35-36)**.

Point 4: "where" after equation Reviewer's Comment: *"The adverb "where" after an equation should be written with small letters."*

Our Response: Thank you for pointing out this detail. We have gone through the entire manuscript and have corrected "Where" to a lowercase "where" after all equations.

Point 5: Comparison with other efficient algorithms Reviewer's Comment: *"- It is essential to compare your algorithm experimentally or through discussion with efficient optimization algorithms such as ICSPM, ICSPM2, Exploratory Cuckoo Search, and*

Improved SSA (ISSA) with HDPM"

Our Response: Thank you for providing these state-of-the-art algorithms for comparison. We have added a new discussion in the **"Related Work" section (Page 6)** acknowledging the importance of these methods. While a direct experimental comparison was beyond the scope of the current study, we have explicitly stated that benchmarking AMTSA against these promising approaches is a valuable direction for future work.

Point 6: Dataset size and cross-validation Reviewer's Comment: *"- The lung cancer dataset (300 images) seems small. Clarify if cross-validation was used to mitigate overfitting."*

Our Response: Thank you for this critical question. To ensure the robustness of our model evaluation and to address your concern about the dataset size, we have now clarified in **Section 6.2 (Page 42)** that we employed a **10-fold stratified cross-validation** strategy to train and evaluate our SVM recognition model. The final reported performance metrics are the average of the 10-fold validation results, which provides a more reliable estimate of the model's generalization ability.

Point 7: Visual examples of segmented images Reviewer's Comment: *"- Include visual examples of segmented CT images (e.g., with 4/10/20 thresholds) to qualitatively validate improvements."*

Our Response: Thank you for this suggestion. To provide more intuitive visual evidence, we have created a **new Figure 20 (Page 40)**. This figure not only displays the segmentation results of our proposed AMTSA method at 4, 10, and 20 thresholds but, importantly, also includes the **original CT image** as a baseline and provides a direct visual comparison with a high-performing competitor, thus more clearly demonstrating the advantages of our method.

Point 8: ST parameter Reviewer's Comment: *"The fixed parameter ST=0.1 is used across all TSA variants. Justify why this value is optimal or discuss sensitivity analysis."*

Our Response: This is a very good point. In response to this and a related comment from Reviewer #1, we have added **Section 5.3, "Parameter Sensitivity Analysis" (Page 32)**. In this section, we conduct a sensitivity analysis on key parameters, including ST. The results indicate that ST=0.1 is a reasonable choice that provides robust performance across a variety of problems.

Point 9: Advantages and disadvantages Reviewer's Comment: *"- What are the advantages and disadvantages of your method over existing methods?"*

Our Response: Thank you for this question. We have added a dedicated discussion in the revised **"Conclusions and Future Work" section (Page 46)**. This paragraph explicitly outlines the **advantages** of AMTSA (e.g., superior optimization performance on complex, high-dimensional problems) and its **disadvantages** (e.g., a moderate increase in computational cost compared to the simple baseline algorithm).

Points 10 & 11: Contributions and Limitations in Conclusion Reviewer's Comment: *"- The contributions of the authors should be described well in the conclusion section. - The limitations of the proposed approach should be mentioned in the conclusion section."*

Our Response: Thank you. We have rewritten the **"Conclusions and Future Work"**

section (Page 46). It now more clearly and explicitly summarizes the three core contributions of this study and significantly expands the discussion on the method's limitations, including those pointed out by you and the other reviewer.
