# Supplementary material for: Adaptive and migration-enhanced tree seed algorithm for multi-threshold CT image segmentation and lung cancer recognition
Source: PLoS One. 2026 Jan 16;21(1):e0333304. doi: 10.1371/journal.pone.0333304 (PMC12810928; doi:10.1371/journal.pone.0333304)
Supplement: Attachment — Submitted filename: Response_to_Reviewers_auresp_2.pdf [file pone.0333304.s002.pdf]

Response to Reviewers

Chenxi Li
Center for Artificial Intelligence,
Jilin University of Finance and Economics,
Changchun 130117, China
Email: : 0314042200902@jlufe.edu.cn
September 7, 2025
To: The Academic Editor, Dr. Mahamed G.H. Omran, and esteemed Reviewers, PLOS ONE
Manuscript ID: PONE-D-25-33941R1
Manuscript Title: Adaptive and Migration-enhanced Tree Seed Algorithm for Multi-Threshold CT Image Segmentation and Lung Cancer Recognition

Dear Dr. Omran and esteemed Reviewers,

Thank you for your thorough review of our manuscript and for providing insightful comments and constructive suggestions. We appreciate the time and effort you have dedicated to our work. We have carefully considered all the points raised and have revised the manuscript accordingly. We believe the manuscript has been significantly improved as a result of these revisions.

Below is our point-by-point response to the comments. All changes have been highlighted in the manuscript file labeled 'Revised Manuscript with Track Changes'.

Responses to the Academic Editor

Editor's Comment 1: Please address the concerns of Reviewer 1.

Our Response: Thank you, Dr. Omran. We have thoroughly addressed all concerns raised by Reviewer #1. A detailed, point-by-point response to each of their comments is provided in the subsequent section. Based on the reviewer's invaluable feedback, we have undertaken a significant revision of the manuscript to enhance its scientific rigor, transparency, and clarity.

Editor's Comment 2: In addition, Please post your code online for the reviewers to check it.

Our Response: Thank you for this requirement. To ensure the reproducibility and transparency of our research, we have made the complete source code for our proposed AMTSA algorithm and the related experiments publicly available. The code can be accessed at the following GitHub repository:**https://github.com/lcxlwxy-droid/AMTSA**. We have also noted in the manuscript that the code will be mirrored at www.jianhuajiang.com upon the paper's official publication.

Responses to Reviewer #1

We sincerely thank you for your detailed and constructive feedback. You noted that while our paper has been seriously improved, several questionable points remained. Your rigorous critique has been invaluable in helping us further elevate the quality and scientific soundness of our manuscript. We have carefully considered each point and have made substantial revisions as detailed below.

Point 1: On the intrinsic quality of the TSA algorithm.

Reviewer's Comment: "As said in my previous review TSA is intrinsically not a good

algorithm. This is because the basic idea is weak... Therefore it is indeed easy to improve it thanks to complicated additional mechanisms, which imply to tune more parameters."

Our Response: Thank you for this insightful perspective. We agree that the native TSA, like many early metaheuristics, has known limitations. We have revised our Introduction to better frame our contribution in this context. Instead of defending the base algorithm, we now explicitly acknowledge its shortcomings (e.g., premature convergence) and position them as the direct motivation for our research. We clarify that our novelty lies not in the TSA framework itself, but in the specific, mechanism-driven enhancements we designed to overcome its inherent deficiencies. This revised framing can be found in the Introduction section, around line 48.

Point 2: On the claim of maintaining high population diversity.

Reviewer's Comment: "line 554 'Compared to TSA, AMTSA not only converges faster, but also maintains a high population diversity throughout the process.' According to Figure 9 not for F1."

Our Response: Thank you for pointing out this contradiction. You are correct; the original statement was an overgeneralization. We have completely rewritten the "Population Diversity Analysis" subsection (around line 548) and revised Figure 9 to include analysis of both unimodal (F1, F2) and multimodal (F8) functions. We now explain that the rapid diversity drop on simple functions like F1 is a desirable sign of efficient exploitation, as the algorithm quickly converges on the single optimum. In contrast, on the complex multimodal function F8, AMTSA actively maintains a significantly higher diversity to facilitate global exploration. This revised analysis provides a more nuanced and accurate account of AMTSA's adaptive diversity control.

Point 3: On the claim of a "dynamic balance" between exploration and exploitation.

Reviewer's Comment: "Also you claim 'a dynamic balance between global exploration and local exploitation' (line 84). But Figure 11 shows that the exploration/exploitation ratio quickly decreases to zero. So what is your formal definition of a 'balance'?" (Also mentioned for line 1041).

Our Response: Thank you for this crucial critique. We agree that the term "balance" was imprecise and not well-supported by Figure 11. We have removed this vague term throughout the manuscript. Instead, we now describe the mechanism more accurately as a "dynamic transition from global exploration to local exploitation." We explain that the algorithm intelligently shifts its focus from exploration in the early stages to exploitation in the later stages, a process clearly illustrated by the trend in Figure 11. This more precise language can be found in the revised "Exploration and Exploitation Analysis" subsection, around line 573, and other relevant parts of the manuscript.

Point 4: On the presentation of convergence curves.

Reviewer's Comment: "Figures 15-17. You present 12 convergence curves on which AMTSA outperforms the other methods. But there are 30 functions in the benchmark. What about the 18 others? If AMTSA is outperformed on some of them you have to say it and to try to explain why."

Our Response: Thank you for this critical point, which highlights the need for a more transparent presentation. To address this, we have revised the text and figures in "Comparative Experiment 2" (around line 636). We now explicitly state that the figures show a deliberately chosen representative set of functions to provide a balanced overview. We have increased the

number of displayed curves and specifically included cases where AMTSA's performance is competitive but not superior (F5, F12, F25, F30), and we analyze these instances in the text. This revision is intended to provide a more comprehensive and objective visual assessment of our algorithm's performance.

Point 5: On the Wilcoxon's test results.

Reviewer's Comment: "Table 10. Result of Wilcoxon's test for AMTSA and other algorithms. The list of algorithms is not the same as in Tables 5-7. In particular CMA-ES does not appear. Why?"

Our Response: Thank you for identifying this serious omission. This was an oversight on our part, and we apologize. We have now re-run the Wilcoxon's signed-rank test to include CMA-ES and all other competitor algorithms from Tables 5-7. The revised Table 10 (around line 749) and its accompanying analysis now provide a complete and fair statistical comparison, which includes cases where the difference is not statistically significant, as discussed in the text.

Responses to Reviewer #2

Reviewer's Comment: "All comments have been addressed... The paper can be accepted for publication."

Our Response: We sincerely thank Reviewer #2 for their positive assessment and for their support of our work. We are very grateful for their time and feedback during the review process.
